# Effect of SGLT2 inhibition on salt-induced hypertension in female Dahl SS rats

**DOI:** 10.1038/s41598-023-46016-z

**Published:** 2023-11-06

**Authors:** Olha Kravtsova, Vladislav Levchenko, Christine A. Klemens, Timo Rieg, Ruisheng Liu, Alexander Staruschenko

**Affiliations:** 1 Department of Molecular Pharmacology and Physiology, University of South Florida, 560 Channelside Dr., Tampa, FL 33602 USA; 2 Hypertension and Kidney Research Center, University of South Florida, Tampa, FL 33602 USA; 3 James A. Haley Veterans’ Hospital, Tampa, FL 33612 USA

**Keywords:** Kidney diseases, Kidney, Hypertension

## Abstract

Sodium-glucose co-transporters (SGLTs) in the kidneys play a pivotal role in glucose reabsorption. Several clinical and population-based studies revealed the beneficial effects of SGLT2 inhibition on hypertension. Recent work from our lab provided significant new insight into the role of SGLT2 inhibition in a non-diabetic model of salt-sensitive hypertension, Dahl salt-sensitive (SS) rats. Dapagliflozin (Dapa) blunted the development of salt-induced hypertension by causing glucosuria and natriuresis without changes in the Renin–Angiotensin–Aldosterone System. However, our initial study used male SS rats only, and the effect of SGLT2 inhibitors on hypertension in females has not been studied. Therefore, the goal of this study was to determine whether SGLT2 inhibition alters blood pressure and kidney function in female Dahl SS rats. The result showed that administration of Dapa for 3 weeks prevented the progression of salt-induced hypertension in female rats, similar to its effects in male SS rats. Diuresis and glucose excretion were significantly increased in Dapa-treated rats. SGLT2 inhibition also significantly attenuated kidney but not heart fibrosis. Despite significant effects on blood pressure, Dapa treatment caused minor changes to electrolyte balance and no effects on kidney and heart weights were observed. Our data suggest that SGLT2 inhibition in a non-diabetic model of salt-sensitive hypertension blunts the development of salt-induced hypertension independent of sex.

## Introduction

Nearly 120 million American adults have hypertension, which is one of the major risk factors for stroke, coronary heart disease, and renal failure^1^. Hypertension, along with diabetes, are the major risk factors for chronic kidney disease (CKD). The prevalence of hypertension tends to be higher in men than in women in younger age groups, but this difference diminishes with age^2^. Around 50% of hypertensive patients are salt sensitive^3^, and recent studies indicate that women are more salt-sensitive than men due to inadequate renin–angiotensin–aldosterone system suppression, excessive aldosterone production, and female sex hormones^4^. Current hypertension treatment guidelines do not consider sex as a variable when prescribing hypertension treatment^5^. Some studies showed that diuretics and calcium channel blockers are more likely to be prescribed for women, while beta blockers and ACE inhibitors are prescribed for men^6,7^.

Sodium-glucose co-transporter 2 (SGLT2) inhibitors (SGLT2i) have demonstrated potential benefits in cardiovascular health. Several clinical and population-based studies revealed the beneficial effects of SGLT2i on hypertension^8,9^. Moreover, approximately half of all hypertensive subjects demonstrate salt sensitivity, with African American populations suffering a disproportionate incidence of salt-sensitive (SS) hypertension^10–12^, with some progressing to end-stage kidney disease (ESKD). However, the effect of SGLT2i on hypertension remains under-explored, particularly in the female population. Female sex as well as a black race, were associated with a lower rate of SGLT2 inhibitor use in the US^13^. Comparing women and men with type 2 diabetes in EMPA-REG OUTCOME, CANVAS Program, DECLARE TIMI-58, and CREDENCE trials showed that both sexes achieve significant cardiovascular protection with SGLT2 inhibition, with no sex differences in the risk reduction^14^. A meta-analysis of cardiovascular outcome trials conducted with SGLT2i in patients with type 2 diabetes with high cardiovascular risk demonstrated a reduction in major adverse cardiovascular events in men but not women^15^.

In this context, the current study aims to investigate the influence of SGLT2i on the development and progression of hypertension in female subjects, using Dahl Salt-Sensitive (SS) rats as our experimental model. Through this study, we intend to provide insights into the role of SGLT2i, specifically dapagliflozin (Dapa), in mitigating hypertension. The outcomes of this research will improve our understanding of the broader cardiovascular implications of SGLT2i and their potential applications in sex-specific hypertension management.

## Results

### Dapa treatment mitigates the progression and intensity of salt-induced hypertension in female Dahl SS rats

**Figure 1:**
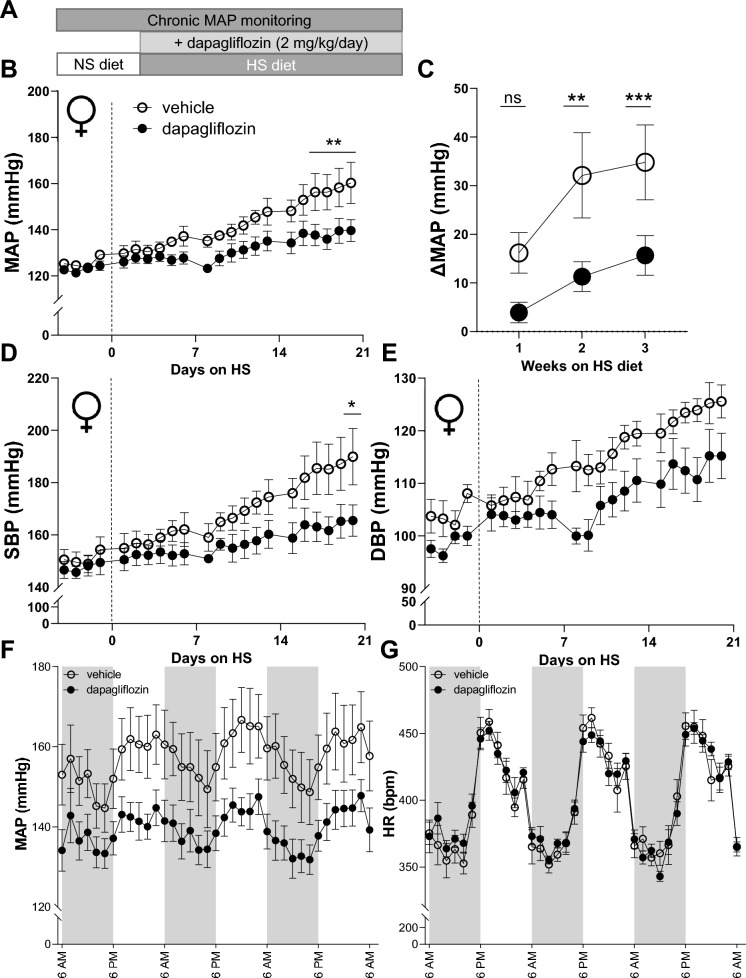
The effect of dapagliflozin treatment on magnitude of blood pressure in female Dahl SS rats. (**A**) Schematic representation of the experimental protocol. (**B**) The mean arterial pressure (MAP) in SS rats after diet change from normal salt (NS, 0.4% NaCl; day 0) to a high salt (HS, 4% NaCl) diet and chronic treatment with the SGLT2 inhibitor (dapagliflozin) or vehicle (**C**) Changes in MAP in weeks 1, 2, and 3 of Dapa treatment on the HS diet compared to baseline MAP on the NS diet. Systolic (SBP) (**D**) and diastolic (DBP) (**E**) blood pressure in SS rats after diet change from normal salt (NS, 0.4% NaCl; day 0) to a high salt (HS, 4% NaCl) diet and chronic treatment with the SGLT2 inhibitor (dapagliflozin) or vehicle. (**F**) Diurnal blood pressure and (**G**) heart rate recordings on HS diet over the last three days, each point is a 2-h average. Shaded regions in (**F**) and (**G**) indicate the active period for animals (lights were off in the animal facility)*.* For all figures n = 6–7, two-way repeated-measures ANOVA was used, *P < 0.05. ** *P* < 0.01, *** *P* < 0.001 are considered statistically significant.

To test an effect of SGLT2i on blood pressure during the progression of salt-induced hypertension, we fed Dahl SS rats with 4.0% NaCl high-salt (HS) diet for three weeks (see protocol in Fig. 1A). At baseline, while the animals were fed a 0.4% NaCl normal salt (NS) diet, the mean arterial blood pressure (MAP) between the vehicle and Dapa groups was not statistically different from each other (129 ± 1 versus 124 ± 2 mmHg, respectively). Exposure to a HS diet for three weeks caused a notable rise in blood pressure, reaching 160 ± 3 mmHg for the group treated with a vehicle but Dapa treatment prevented the increase to levels seen in vehicle treatment (139 ± 7 mmHg) (*P* < 0.05). Throughout the final week of the study (D13-D21), the Dapa-treated rats displayed a markedly reduced blood pressure, maintaining ~ 20 mmHg lower by the end of the experiment (Fig. 1B and C; D21, *P* < 0.001). A similar pattern was seen for systolic (SBP) and diastolic blood pressure (DBP) measurements when comparing Dapa-treated to vehicle-treated rats (Fig. 1D and E); however, the statistically only SBP was different at the end of the experiment. No differences in circadian variations of MAP (Fig. 1F) and heart rate (Fig. 1G) were observed between groups.

**Figure 2:**
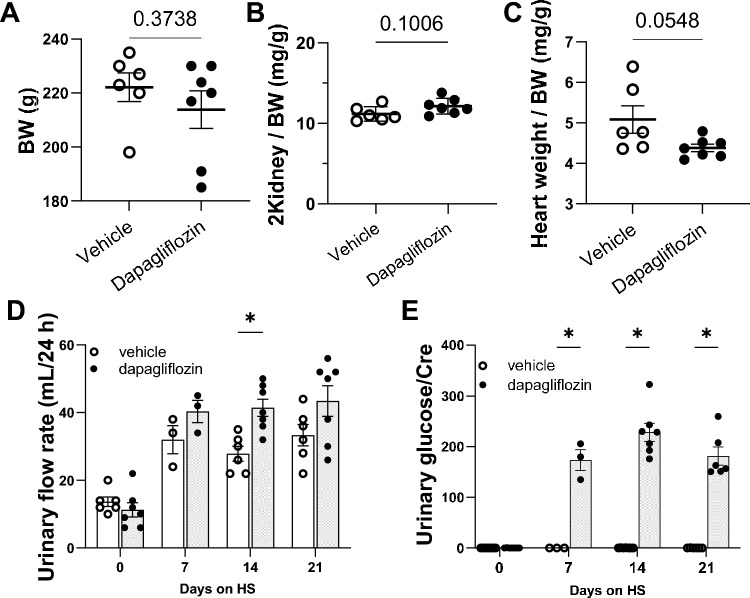
Body composition, diuresis and glucosuria changes under dapagliflozin treatment in female Dahl SS rats. (**A**) body weight (BW), (**B**) normalized 2 kidney weight to BW, (**C**) normalized heart weight to BW. (**D**) Twenty four hour urinary flow rate of Dahl SS rats at days 0, 7, 14, and 21. (**E**) Urinary glucose concentrations at days 0, 7, 14, and 21. n = 6–7. Data were compared using Student’s *t*-test. * *P* < 0.05. ** *P* < 0.01, *** *P* < 0.001 are considered statistically significant. For day 7 only 3 data points are shown due to the technical issues during the urine collection.

Kidney and heart weights were analyzed to determine if Dapa had an effect on organ weights. No significant difference was found in body weight between the control and experimental groups (Fig. 2A). Also, the ratio of kidney weight to body weight (2Kidney/BW) was not different in the Dapa-treated group compared to the vehicle-treated rats at the end of the experiment (Fig. 2B). Dapa-treated rats showed a significantly lower heart weight; however, this difference was lost when comparing heart weight to body weight ratios (Fig. 2C).

Consistent with the pharmacodynamics of SGLT the administration of Dapa increased glucose excretion. This was associated with ~ two-fold greater diuretic effect compared to vehicle on D14 (*P* < 0.05, Fig. 2D). Moreover, urinary glucose concentration normalized to urinary creatinine and absolute glucose excretion were significantly elevated in Dapa-treated rats to vehicle-treated (182 ± 18 versus 0.3 ± 0.03 mg/dL and 101 ± 17 versus 0.18 ± 0.03 mg/day on D21, respectively, *P* < 0.001; Fig. 2E).

### Dapa treatment does not affect electrolyte homeostasis in female rats

**Figure 3:**
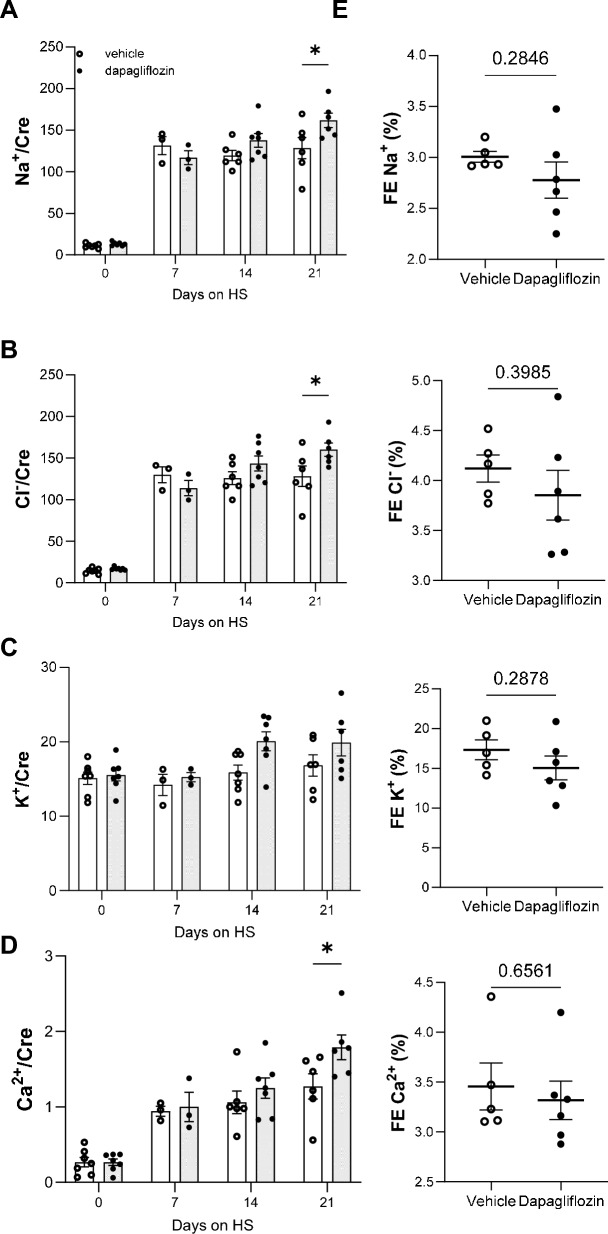
Urinary electrolytes normalized to creatinine in female SS rats in response to dapagliflozin treatment. Electrolytes at the days 0, 7, 14, 21: (**A**) sodium, (**B**) chloride, (**C**) potassium and (**D**) calcium. (**E**) Fractional excretion (FE) of Na^+^, Cl^−^, K^+^, Ca^2+^, Mg^2+^, PO_4_^3−^, and uric acid over 24 h in vehicle- and Dapa-treated Dahl SS rats, *P*-values are given for each graph, *P < 0.05. n = 6–7. Data were compared using Student’s *t*-test.

Dapa treatment did not alter the urinary electrolytes to creatinine ratios on D7, D14 compared to a vehicle (Fig. 3A–D). However, Na^+^, Cl^−^ and Ca^2+^ excretion were significantly higher in Dapa-treated rats at the end of the experimental period (Fig. 3A, B and D, respectively). No significant differences in urinary K^+^ to creatinine ratios were observed between the groups (Fig. 3C). An estimation of fractional excretion showed no differences between groups (Na^+^, K^+^, Cl^−^, Ca^2+)^ (Fig. 3E). By the end of the experimental protocol, no significant changes were observed between groups in plasma electrolytes and creatinine and non-fasting blood glucose (Table 1). Fasting blood glucose levels were not measured to avoid changes in blood pressure due to caloric restriction.

**Table 1:** Blood parameters in female SS rats after 21 days of chronic experiment with dapagliflozin treatment on HS.

	Vehicle	Dapagliflozin
Na^+^ (mM)	135 ± 2	135 ± 3
K^+^ (mM)	3.0 ± 0.2	3.1 ± 0.1
Ca^+^ (mM)	1.21 ± 0.02	1.23 ± 0.03
Cl^−^ (mM)	103 ± 3.7	97 ± 1.6
Glu (mM)	297 ± 16	286 ± 22
Creatinine (mg/dL)	0.31 ± 0.04	0.26 ± 0.01

### Dapa treatment does not affect creatinine clearance but attenuates kidney fibrosis in female rats

**Figure 4:**
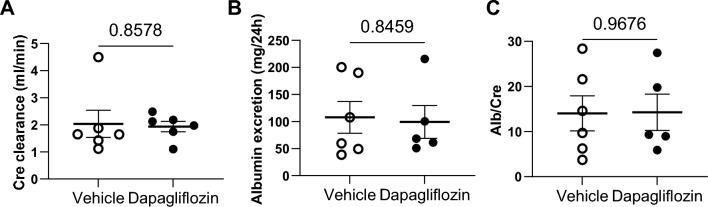
Creatinine clearance and albuminuria in female Dahl SS rats under dapagliflozin treatment. (**A**) Creatinine clearance, (**B**) albumin excretion, (**C**) Albumin-to-Creatinine ratio. n = 6–7. Data were compared using Student’s *t*-test.

**Figure 5:**
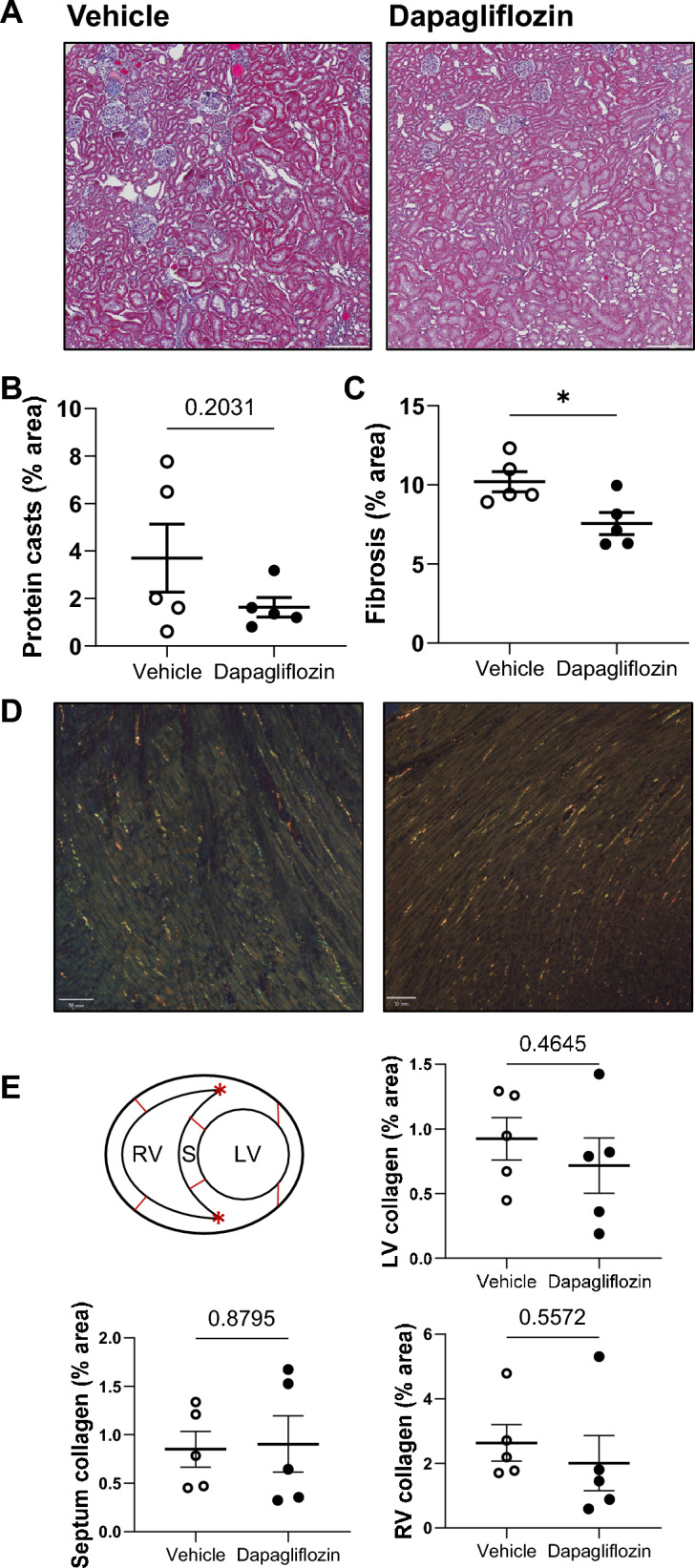
Changes in kidney and heart damage in female Dahl SS rats under dapagliflozin treatment. (**A**) Representative images of kidney tissue stained with Masson’s trichrome at 10×  agnification. Scale bars are 200 µm. Summary graphs of the (**B**) medullary protein casts area (percent total kidney area) and (**C**) whole kidney fibrosis. (**D**) Representative images of Picrosirius Red stained heart sections for fibrosis detection at magnification 10×. Scale bars are shown. (**E**) Summary graph of heart fibrosis (LV—left ventricle, RV—right ventricle, S—septum). * *P* < 0.05. N = 5. Data were compared using Student’s *t*-test.

Subsequently we examined whether the beneficial effect of Dapa treatment on MAP resulted in concurrent effects on kidney function and damage. Creatinine clearance did not show significant differences between Dapa and vehicle groups (Fig. 4A). Consistent with this, urinary albumin excretion (Fig. 4B), as well as urinary albumin-to-creatinine ratio (Fig. 4C) and plasma creatinine (Table 1), did not show significant differences between groups. Subsequently, we evaluated the influence of SGLT2 inhibition on structural kidney damage. Examination of Masson’s Trichrome stained kidney sections showed that protein casts did not vary significantly between the groups. However, semiquantitative analysis showed lower cortical fibrosis in the treated group (Fig. 5A–C).

### Dapa treatment does not affect the amount of collagen in the hearts of female rats

To evaluate the degree of myocardial collagen, we used Picrosirius Red staining imaged with polarized light to highlight collagen and selected multiple frames from different cardiac regions. This analysis of stained sections showed that there was no significant difference in the left, right ventricular or septum cardiac connective tissue amount between Dapa- and vehicle-treated rats (Fig. 5D, E).

## Discussion

In the current study, we determined the effects of the SGLT2i Dapa on the development of hypertension and severity of target-organ damage in female Dahl SS rats. This study adds valuable information and complements our previous studies, which predominantly focused on male rats^16^. It is known that animal models of salt-sensitive hypertension exhibit lower blood pressure in females than in males^5–7^. Here we show that Dapa administration to female rats did not differ significantly from males in the magnitude of blood pressure reduction, which we reported previously^16^. The rationale for completing these experiments is due to known sex differences in renal transporter expression^17^. Differences in SGLT2 expression were observed between female and male rodents. In C57BL/6 mice, SGLT2 protein expression is lower in females compared to males; however, *Sglt2* mRNA expression showed the opposite^18^. In contrast, in Wistar rats, SGLT2 protein expression was higher in females compared to males^18,19^ and *Sglt2* mRNA expression in the renal cortex was similar^18^. In human kidneys no differences in SGLT2 expression were reported^20^.

While SGLT2 is not a major contributor to sodium transport in the kidneys, its inhibition was associated with several pleiotropic effects on electrolytes in distal nephron segments, particularly in the distal convoluted tubule and the collecting duct. Female rats have more rapid diuretic and natriuretic responses to saline load, possibly due to differences in transporter expression in proximal and distal nephrons^17^. Veiras et al. found that compared to male rats, female rats have increased phosphorylation and localization of NHE3, lower expression of Na^+^-P_i_ cotransporter 2, aquaporin-1, and claudin-2 in the proximal tubule together with lower Na^+^ and HCO_3_^−^ reabsorption and therefore increased flow from out of the proximal tubule. In distal segments of the nephron, female rats have a higher abundance of total and phosphorylated Na^+^-Cl^−^ cotransporter (NCC), claudin-7, and cleaved forms of α- and γ-ENaC subunits^17^. At variance to our findings in male Dahl SS rats^16^, the diuretic effect of Dapa was less pronounced in females. If this relates to differences in SGLT2 expression or differences in the compensatory responses in further downstream segments remains to be determined.

Interestingly, one of the major differences we observed between males and females that were treated with Dapa was the kidney-to-body weight ratios. In the male treatment group, there was a significant increase in the kidney-to-body weight ratios^16^. In contrast, the Dapa-treated females did not exhibit this change. It has been proposed that SGLT2i treatment can increase kidney weights by increasing the cellular area of the S3 proximal tubule and collecting duct nephron segments^21^. This study, however, only investigated this change in male mice, and not females. It would be of additional physiological significance to look into this sex difference more closely in the future, as it may be of clinical interest.

Our results demonstrate that Dapa administration reduced the magnitude of blood pressure increase in female salt-sensitive rats without renal electrolyte handling or kidney hypertrophy. Retrospectively comparing current results to the previous study in male^16^, female Dahl SS rats showed the same amplitude in blood pressure changes under Dapa treatment (~ 20 mmHg) despite the similar MAP pressure (Table 2). This data suggests that dapagliflozin blunts salt-sensitive hypertension both in male and female rats. Studies investigating the effect of SGLT2i on blood pressure and renal function in female subjects are limited but have provided some insight into their effects in females. In one study, empagliflozin treatment of hyperandrogenic female rats (polycystic ovary syndrome model) showed only a small decrease in blood pressure, and no effect on albuminuria was observed^22^. In female db/db mice, empagliflozin improved diastolic function, reduce cardiomyocyte cross-sectional area, myocardial fibrosis, and pro-fibrotic SGK1/ENaC protein expression levels but not blood pressure or dipping status^23^. It is worth noting that SGLT2i also have potential implications for the treatment of preeclampsia. One study employing AT1-AA-induced preeclampsia in mice showed that empagliflozin might have a protective effect on preeclampsia by ameliorating systolic blood pressure, proteinuria, podocyte injury and oxidative stress^24^.

**Table 2:** Comparing of the effect of dapagliflozin on blood pressure in male and female SS rats.

MAP, mmHg	Male*^16^	Female
Baseline (day-3), NS, vehicle group	119 ± 2	124 ± 1
Baseline (day-3), NS, treated group	120 ± 2	123 ± 2
HS, day 21, vehicle group	156 ± 6	160 ± 9
HS, day 21, treated group	136 ± 3	140 ± 5
Δ day 21, HS	20	20

*Experiments were performed at different time, retrospective data for male Dahl SS^16^ was used.

The renoprotective properties of SGLT2i are well-established. Numerous preclinical and clinical studies have highlighted the connection between SGLT2i treatment and a decrease in various markers of inflammation and kidney injury^25,26^, as well as preservation of kidney function decline in CKD^27–29^. Animal-based research has also indicated the renoprotective effects of gliflozins^30–33^. In Angiotensin II-dependent hypertension, empagliflozin administration prevented the development of renal fibrosis without affecting blood pressure^34^. Empagliflozin also suppressed kidney fibrosis, renal growth and inflammation in diabetic mice^33,35^. The beneficial role of SGLT2i was also shown to reduce the risk of heart failure progression or mortality in patients regardless of the presence of diabetes mellitus^36^. Our experiments showed a noticeable decrease in cortical fibrosis but not protein cast development after Dapa treatment in female Dahl SS rats. However, no specific improvements to cardiac fibrosis or heart rate were observed.

In summary, our findings demonstrate that administration of Dapa prevents salt-induced hypertension in female rats and reduces renal cortical tissue fibrosis but does not affect electrolyte excretion or cardiac damage. Our findings thus highlight the importance of including sex as a biological variable in pharmacological research. This factor is particularly important in studies involving diseases that affect men and women differently.

## Methods

### Experimental protocol and animals

Animal experiments and procedures adhered to the National Institutes of Health (NIH) Guide for the Care and Use of Laboratory Animals and reported in accordance with ARRIVE guidelines. The protocols were reviewed and approved by the University of South Florida (USF) Institutional Animal Care and Use Committee. Female Dahl salt-sensitive rats (SS; SS/JrHsdMcwi; RRID: RGD_61499) were fed normal salt (NS) diet (0.4% NaCl; #113755; Diets Inc., New Brunswick, NJ). For the high salt challenge, rats were placed on 4% NaCl high salt (HS) diet (D113756; Diets Inc.). Rats were maintained on an HS diet for 3 weeks and treated with Dapa or vehicle. Dapa (Cat. 464620010; ThermoFisher Scientific, Waltham, MA) was added to the drinking water at ~ 2 mg/kg/day (the stock solution was prepared in 100% ethanol in a concentration of 20 mg/ml). The concentration of the drug in water was adjusted with the increased water consumption to keep a constant dose during the experiment. Water and food were provided ad libitum. The treatment was initiated on the first day of the HS challenge, as shown in previously published protocols^16^.

### Surgical procedures

At the age of 8–8.5 weeks, rats were anesthetized on a temperature-controlled platform via inhalation of 2.5% isoflurane in 0.5 L/min (O_2_/N_2_: 30%/70%). A blood pressure transmitter (HD-S10; Data Sciences International, New Brighton, MN) was implanted subcutaneously. The catheter tip was secured in the abdominal aorta via the femoral artery^37,38^. Rats were allowed to recover for 4–5 days, and blood pressure and heart rate were recorded using DSI software. At the end of the experiment timeline, rats were anesthetized by 5% isoflurane, and kidneys were flushed with phosphate-buffered saline via aortic catheterization^39–41^. The left kidney was snap-frozen, and the right kidney was placed in 10% formalin for histological studies.

### Electrolyte measurements and albumin assay

Urine was collected for 24 h in metabolic cages (no. 37000M071, Tecniplast, West Chester, PA) at baseline and every 7 days of the HS protocol. Prior to euthanasia, blood samples were collected by aortic catheterization in anesthetized animals. Glucose, creatinine, and electrolytes (Na^+^, K^+^, Ca^2+^, Cl^−^) in plasma and urine were measured with a blood gas analyzer (ABL system 800 Flex, Radiometer, Copenhagen, Denmark). Fractional excretion (FE) of electrolytes was calculated by the formula FEX (%) = [Urine electrolyte concentration (mmol/L, or mg/dL) × serum creatinine (mg/dL) divided by serum electrolyte concentration (mmol/L, or mg/dL) × urine creatinine (mg/dL)] × 100%, where the variable ‘X’ represents the specific electrolyte under consideration. Urine albumin was determined by a fluorescent assay (Albumin Blue 580 dye, Molecular Probes, Eugene, OR) using a fluorescent plate reader (Synergy Neo 2, Bio-Tek, Winooski, VT).

### Histological analysis

Rat kidneys and hearts were formalin-fixed, paraffin-embedded, sectioned, and mounted on slides as previously described^41,42^. Slides were processed with Masson’s trichrome stain. Protein cast and fibrosis analysis were assessed in whole kidney slides scanned at × 10 magnification using color deconvolution and thresholding in QuPath-0.4.2 software^43^. Fibrosis was quantified for the cortex area, and protein casts for the outer medulla area. Collagen fibers in the heart tissue sections were visualized using Picrosirius Red staining under polarized light microscopy. For semi-quantification of collagen fibers, each region-of-interest was drawn on the middle third of the free walls of both ventricles (right (RV) and left ventricle (LV) and septum (S) based on the inner junctional points (red asterisk) (Fig. 5E).

### Statistics

Statistical analysis was performed following recent guidelines^44^. The data are displayed as the mean ± the standard error of the mean (SEM). In the box plot illustrations, the box signifies ± SEM. The data underwent tests for normality using the Shapiro–Wilk method and for equal variance via Levene’s homogeneity test. Statistical evaluations were carried out using ANOVA or t-tests (GraphPad Prism 9.0), considering a *P*-value of less than 0.05 to indicate significance. Moreover, if the results of an ANOVA test were significant, a post hoc analysis was conducted using Holm-Sidak's multiple-comparison method.

## Data Availability

All data generated or analyzed during this study are included in this published article.
